# Hydrothermal Synthesis of Cellulose Nanocrystal-Grafted-Acrylic Acid Aerogels with Superabsorbent Properties

**DOI:** 10.3390/polym10101168

**Published:** 2018-10-19

**Authors:** Xuehua Liu, Rue Yang, Mincong Xu, Chunhui Ma, Wei Li, Yu Yin, Qiongtao Huang, Yiqiang Wu, Jian Li, Shouxin Liu

**Affiliations:** 1 Key Laboratory of Bio-Based Material Science and Technology of Ministry of Education, Northeast Forestry University, Hexing Road 26, Harbin 150040, China; aliuxuehua@126.com (X.L.); m18846145637@163.com (M.X.); mchmchmchmch@163.com (C.M.); nefuyinyu@163.com (Y.Y.); nefulijian@163.com (J.L.); 2 Post-Doctoral Research Center, Yihua Lifestyle Technology Co., Ltd., Shantou 515834, China; yangre@yihua.com (R.Y.); huangqt@yihua.com (Q.H.); 3 School of Materials Science and Engineering, Central South University of Forestry and Technology, Changsha 410004, Hunan, China; wuyq0506@126.com

**Keywords:** cellulose nanocrystals, hydrothermal, aerogels, superabsorbent

## Abstract

In this work, we applied a fast and simple method to synthesize cellulose nanocrystal (CNC) aerogels, via a hydrothermal strategy followed by freeze drying. The characteristics and morphology of the obtained CNC-g-AA aerogels were affected by the hydrothermal treatment time, volume of added AA (acrylic acid), and the mass fraction of the CNCs. The formation mechanism of the aerogels involved free radical graft copolymerization of AA and CNCs with the cross-linker *N*,*N*′-methylene bis(acrylamide) (MBA) during the hydrothermal process. The swelling ratio of the CNC-g-AA aerogels was as high as 495:1, which is considerably greater than that of other polysaccharide-g-AA aerogels systems. Moreover, the CNC-g-AA aerogels exhibited an excellent methyl blue (MB) adsorption capacity and the ability to undergo rapid desorption/regeneration. The maximum adsorption capacity of the CNC-g-AA aerogels for MB was greater than 400 mg/g. Excellent regeneration performance further indicates the promise of our CNC-g-AA aerogels as an adsorbent for applications in environmental remediation.

## 1. Introduction

Developments in industry and commerce have raised the problem of dye contamination, which is characterized by a high organic matter content, resistance to biodegradability, and complex biological toxicity [1]. Dye contamination raises serious environmental concern with potential dangers to food safety and aquatic life [2]. Various groups have tried to develop more efficient methods of removing dye pollutants. Dye pollution removal is conventionally achieved by photochemical degradation, membrane filtration, flocculation, biological oxidation, and chemical precipitation [3,4]. However, these technologies are complex and expensive; hence, the design and development of alternative low-cost adsorbents has been the focus of many studies.

Cellulose nanocrystals (CNCs) are a renewable material with properties including good biodegradability, mechanical strength [5], flexibility [6], easily modified, and biocompatibility [7,8]. The high strength, dimensional anisotropy, and natural sourcing of CNCs, have attracted considerable research interest for their use as functional and renewable reinforcing agents for polymer composites [9,10]. The rigid and flexible mechanical properties of CNCs show great potential for various applications and have motivated research on preparing CNC aerogels for different fields [11,12,13]. The fabrication of CNC-based aerogels can avoid issues related to cellulose gel shrinkage, capillary tension, and rupturing in drying processes owing to capillary pressure [14]. Functionalized CNC aerogels can exhibit multiple responses according to changes in external conditions, such as temperature, pH, and pressure [15,16]. Thus, CNC aerogels can be used in biological applications and as strained materials, adsorbent materials, hydrophobic materials, and conductive materials. There are also ideal materials for applications in environmental modification [17,18,19].

Highly porous CNC-based aerogels with many hydroxyl groups are widely regarded as superabsorbent materials [20]. The unique properties of these materials include high strength, high surface area, and tunable surface chemistry, which enable interactions with other polymers. In particular, functional polymeric materials, containing a strong hydrophilic group introduced by graft copolymerization reactions, are widely used in polysaccharide reaction systems because of their high efficiency and compatibility. CNC-based aerogels can be prepared by a free chain graft polymerization with poly-methacrylic acid, which is modified by functionalizing the carboxylic group [21]. CNC-based aerogels can also be prepared by free radical polymerization of poly(*N*-isopropylacrylamide) (PNIPAM) and grafted by copolymerization of poly (acrylic acid) (PAA), to achieve functionalization by cooperative hydrogen bonding interactions between PNIPAM or PAA and the CNCs [16,22]. Moreover, CNCs, which are graft-copolymerized with AA (acrylic acid) give hydrogels with a swelling ratio far greater than that of other polysaccharide systems combined with AA hydrogels, at the same initial reactant ratio [23,24].

Recently, hydrothermal procedures to produce hydrogels from cellulosic biomass have received attention owing to their inherent advantages in terms of liquid chemical reactions and their simplicity [25]. Compared with reactions at ambient temperature and pressure, the straight forward hydrothermal method developed here requires less reagents to promote the reaction to completion. Our group has prepared fluorescent CNC/carbon dot hydrogels by a one-step hydrothermal method [26]. The shape and chemical structure of the products can also be controlled by the hydrothermal method. Hydrothermal methods are considered to be highly effective for fabricating CNC-based hydrogels [27,28]. These results encouraged us to use a hydrothermal strategy to promote a graft-copolymerized reaction to synthesize a CNC-based aerogel superabsorbent.

In this study, we proposed a simple method to fabricate CNC-g-AA aerogels via a hydrothermal treatment followed by freeze drying. We examined the swelling performance of the obtained aerogels for the water absorption and the adsorption properties of the CNC-g-AA aerogels for methylene blue (MB).

## 2. Materials and Methods

### 2.1. Materials

The CNCs were prepared from commercial bleached kraft softwood pulp (85% International Standardization Organization brightness) as the raw material, which was provided by a paper-making factory in Heilongjiang, China. Analytical-grade sulfuric acid was purchased from Tianjin Kermel Chemical Reagent Co., Ltd. (Tianjin, China). Tetramethylethylenediamine (TMEDA), *N*,*N*′-methylene bis(acrylamide) (MBA), and ammonium persulfate (APS) were purchased from Aladdin Reagent Corp China (Chengdu, Sichuan Province, China). The AA and MB were purchased from Tianjin Fuchen Chemical Reagent Factory (Tianjin, China). All chemical reagents were of analytical grade.

### 2.2. Preparation of CNC-g-AA Hydro/Aerogels

An aqueous CNCs suspension was prepared by a sulfuric acid hydrolysis method, as previously reported [29,30]. Bleached kraft softwood was first milled and then passed through a 0.5-mm screen to ensure a uniform particle size. Then, the milled pulp was hydrolyzed in sulfuric acid (64 wt %; 8.75 mL of sulfuric acid solution per gram of pulp) at 45 °C for 30 min with vigorous stirring. The cellulose suspension was then diluted, centrifuged, washed, and dialyzed against distilled water until the exterior water became neutral before further use.

The CNC-g-AA hydrogels were prepared by a free radical graft copolymerization of CNCs and AA in the presence of a cross-linker (MBA) and a redox initiator system (APS/TMEDA). A 10 mL portion of the prepared CNC solution (1.5 wt %) was placed in a 100 mL three-necked flask, which was then placed in a 25 °C water bath and stirred with a magnetic stirrer. A 0.050 g portion of APS and 0.050 mL of TMEDA, as an initiator, were added to the solution and the mixture was stirred for 10 min to generate radicals. Thereafter, a cross-linker, MBA (0.080 g) and a certain volume of AA were added, followed by continuous stirring for 2 h. The reaction solution was then placed in a Teflon-lined stainless steel autoclave (100 mL) and heated to the desired temperature. The hydrothermal reaction was performed for a specific time. The obtained gels were washed in distilled water to remove unreacted substances.

**Figure 1:**
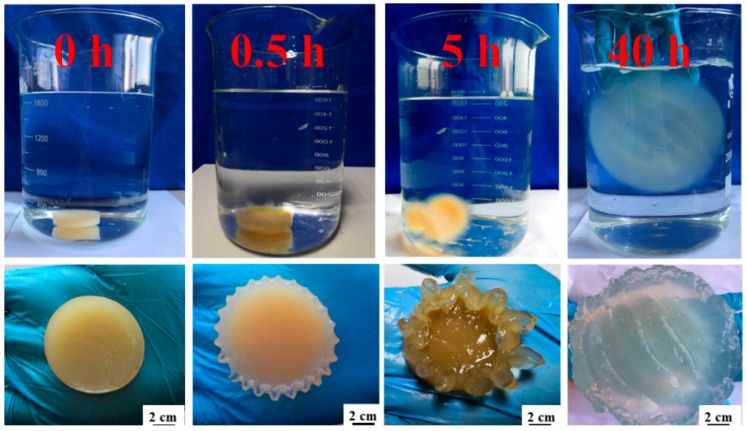
Photographs showing swelling and size-change of CNC-g-AA-2-120-8 gel in distilled water over time.

Thereafter, the as-prepared hydrogels were frozen in liquid nitrogen. After complete freezing, the frozen samples were subjected to freeze-drying (Scientz-10N, Ningbo, Zhejiang Province, China) to obtain pristine CNCs-g-AA aerogels. The resulting CNC-g-AA hydro/aerogels are referred to as CNC-g-AA-r-m-n, where r denotes the AA content, m denotes the hydrothermal temperature, and n denotes the hydrothermal time. Furthermore, we prepared pure hydro/aerogels, where the CNCs solution was replaced with distilled water under conditions of 2.0 mL AA, a hydrothermal temperature of 120 °C, and a treatment time of 8 h (D-g-AA-2-120-8 hydro/aerogels). Herein, the swelling of CNC-g-AA-2-120-8 gel performance in distilled water is shown in Figure 1.

### 2.3. Characterization of CNC-g-AA Aerogels

FT-IR spectra of CNC-g-AA aerogels were measured on a Fourier transform infrared spectrometer (iS10, Nicolet, Thermo Scientific, Waltham, MA, USA) over the range of 400–4000 cm^−1^ in transmission mode. For morphological studies, we examined the CNC-g-AA aerogels with a scanning electron microscope (SEM, QUANTA200, FEI, Hillsboro, OR, USA). Specimens were coated with gold before SEM observations. The ^13^C CP/MAS NMR analysis of the samples was performed at room temperature with a Bruker DRX-400 spectrometer. Spectra were acquired with a 4-mm MAS probe using a combination of CP, MAS, and high-power proton decoupling methods. A total of 800 scans were accumulated for each sample.

### 2.4. Swelling Characterization of CNC-g-AA Aerogels in Distilled Water

The equilibrium swelling ratios of the CNC-g-AA aerogels were measured in distilled water at room temperature. A portion of the CNC-g-AA aerogels (W_1_) was then prepared and immersed in an excess of distilled water to achieve a state of equilibrium swelling. The weight gain of the equilibrium samples (W_2_) was monitored gravimetrically. Excess water on the surface of the hydrogels was removed with filter paper. Three replicates were performed for each composition. The swelling ratio was calculated by the following Equation:

(1) $$\mathrm{SR}=\frac{\left(w_{2}-w_{1}\right)}{w_{1}}$$

### 2.5. Adsorption Studies

We selected the CNC-g-AA-2-120-8 aerogel for the adsorption experiments under different experimental conditions. All experiments were performed in glass vials placed in a controlled-temperature water bath oscillator operating at 150 rpm. The MB solutions of different concentrations were prepared with distilled water. The pH of the dye solutions was adjusted with 0.01 M HCl or NaOH. After dye adsorption equilibrium was reached, we filtered the swollen gels and the residual concentration of filtered MB solution was measured with a UV-vis spectrophotometer (Evolution 600, Thermo Scientific Inc., Madison, WI, USA) and calculated from the absorption intensity at 664 nm. The amount of adsorbed MB was calculated based on the following Equation:

(2) qe = (c0−ce)vm

where q_e_ (mg/g) is the amount of the adsorbed dye at equilibrium; c_0_ (mg/L) and $c_{e}$ (mg/L) are the initial and residual concentrations of the dye solution at equilibrium, respectively; V (L) is the volume of the dye solution; and m (g) is the mass of the adsorbent used.

We selected CNC-g-AA-2-120-8 aerogel for regeneration testing under conditions of MB solution of 20 mg/L, pH = 8.0, adsorbent mass is 0.0050 g. Here, HCl (1 mol/L) was used as the eluent. The CNC-g-AA-2-120-8 hydrogel was loaded with MB and treated with the eluent (50 mL) for 120 min at room temperature. The desorbed adsorbent was washed, freeze-dried, and prepared for the next cycle of regeneration testing.

## 3. Results and Discussion

### 3.1. Morphology of CNC-g-AA Aerogels

**Figure 2:**
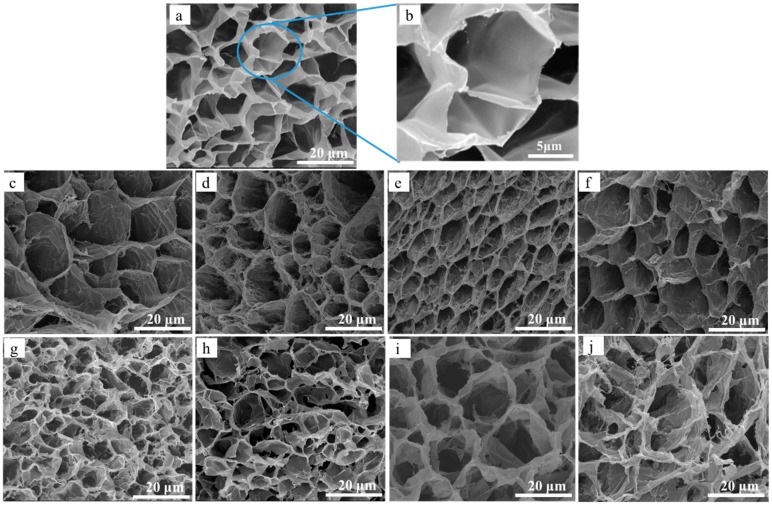
SEM images of (**a**,**b**) D-g-AA-2-120-8 aerogel; (**c**–**f**) CNC-g-AA-r-120-8 aerogels prepared with different AA contents (1, 2, 3, and 4 mL); (**g**–**j**) CNC-g-AA-2-120-n aerogels with different hydrothermal treatment times (2, 4, 8, and 12 h).

The SEM images of D-g-AA aerogel and CNC-g-AA-r-m-n aerogels (Figure 2) showed three-dimensional interconnected honeycomb open-cell structures. The cell wall surfaces of D-g-AA aerogel, without added CNCs, were smooth (Figure 2a,b). For the aerogels with added CNCs, the surface of the aerogel cell walls became wrinkled. Additionally, some web-like structures formed on the cell walls, which likely improved the accessibility of water into the cell. The effects of the hydrothermal treatment time might be related to the CNCs having many active –OH groups and modified –COOH groups on their surfaces, which affected the polymerization of the composites and the formation of the three-dimensional polymer network [31,32]. For longer hydrothermal treatment times, -CO-NH- in MBA tended to combine with carboxyl groups, which contributed to the formation of a three-dimensional honeycomb network [33]. This extended dislocation structure was caused by vacancy motion at the nanoscale in the mixed liquid crystal lattice, which is conducive to the formation of a three-dimensional macroporous structure in the CNCs mixture [34,35]. This type of chemical modification can increase the hydrophilicity of CNCs and their compatibility with hydrophilic polymer matrices. Pores are regions where water can permeate, which allows interactions of external compounds with the hydrophilic groups of the graft copolymers [36].

### 3.2. FT-IR and NMR Characterization of CNC-g-AA Aerogels

**Figure 3:**
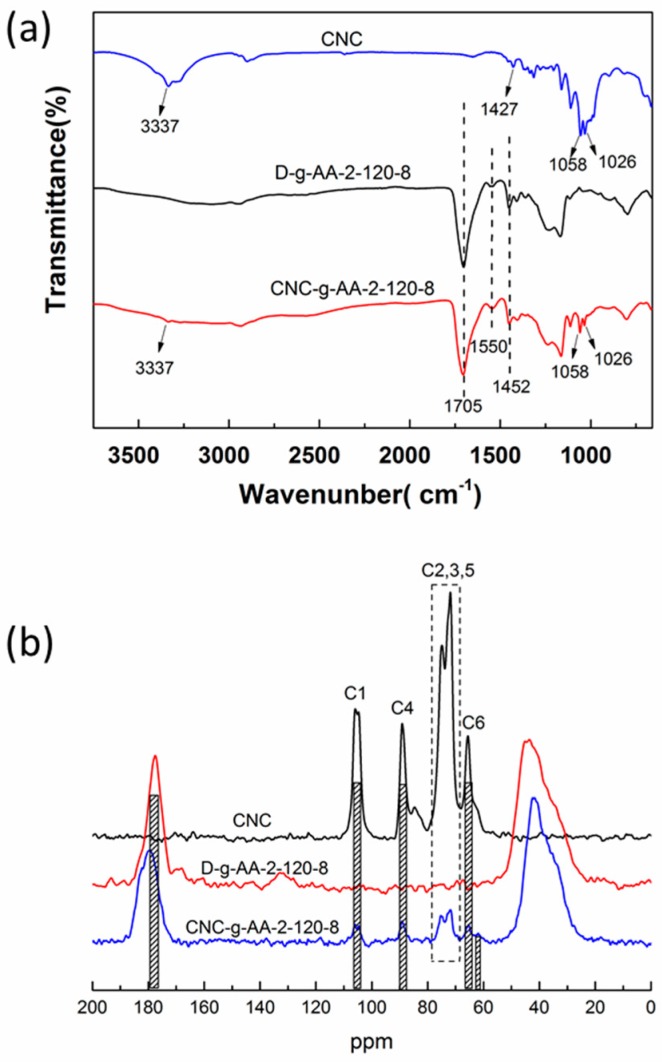
FT-IR (**a**) and ^13^C NMR (**b**) spectra of CNC, D-g-AA-2-120-8 aerogels and CNCs-g-AA-2-120-8 aerogels.

FT-IR spectra of pure CNCs, D-g-AA-2-120-8 aerogel, and CNC-g-AA-2-120-8 aerogel, are shown in Figure 3a. In spectrum a, peaks at 1058, 1427 and 3337 cm^−1^ are associated with CNCs [37]. The peaks at 3337 and 1026 cm^−1^ correspond to the O–H stretching vibration and deformation vibration peaks, indicating strong hydrogen-bonding interactions of CNCs [38]. The region of low intensity bands between 1058 and 1427 cm^−1^ suggests the presence of C–O and C–H bonds [39]. In spectra b and c, we assign the peak at 1550 cm^−1^ to the stretching vibration of N–H. The sharp band at 1705 cm^−1^ is assigned to a symmetrical stretching vibration of COO^−^, which indicated the presence of COO^−^ groups in the CNC-g-AA-2-120-8 aerogel network. These results confirmed that the AA monomers were successfully grafted onto the backbone of CNCs and confirmed our interpretation of the formation of the three-dimensional network structure.

To further illuminate the structure of CNC, D-g-AA and CNC-g-AA, the samples were characterized by ^13^C CP/MAS NMR spectroscopy (Figure 3b). The spectra of CNC displayed the typical signals from cellulose, which were assigned as follows: the C1 (104.7 ppm), C2/C3/C5 (71.9 and 74.9 ppm), C4 (89.1 ppm), and C6 (65.6 ppm) peaks correspond to the carbon atoms of the glucopyranose rings in the crystalline regions, whereas the C4 (84.7 ppm) peak corresponds to those in the amorphous areas [40]. For CNC-g-AA-2-120-8, the peak at 177.5 ppm is associated with carbon atoms of carboxylic acid groups, the peak at 61.9 ppm for N–CH_2_–CH_2_–O– which supports the occurrence of grafting reaction between the hydroxyl groups of CNC and MBA. So, it is apparent that the poly (acrylic acid) chains have been successfully cross-linked on CNC in the presence of the MBA cross-linker [41].

### 3.3. Equilibrium Swelling Ratio in Distilled Water

**Figure 4:**
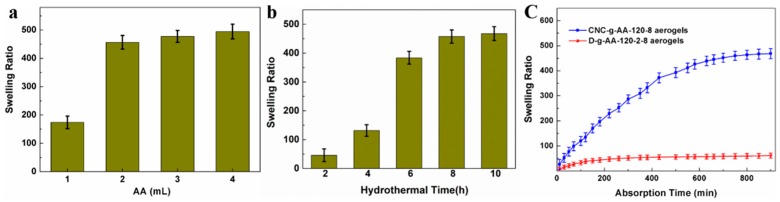
(**a**) Swelling ratio of CNC-g-AA-r-120-8 aerogels with different AA contents; (**b**) Swelling ratio of CNC-g-AA-2-120-n aerogels hydrothermally treated for different times; (**c**) Adsorption capacity of D-g-AA-2-120-8 aerogels and CNC-g-AA-2-120-8 aerogels compared with the adsorption time.

The equilibrium swelling ratio of the CNC-g-AA-r-120-n aerogels was also related to AA content and hydrothermal reaction time (Figure 4a,b). As the amount of added AA was increased from 1.0 to 4.0 mL, the swelling ratio of the CNC-g-AA-r-120-8 aerogels increased from 174 to 495. As the hydrothermal reaction time was increased from 2 to 10 h, the swelling ratio of the resulting aerogels increased from 46 to 478. Hence, we selected the CNC-g-AA-2-120-8 aerogels for detailed analysis of their water and MB adsorption properties.

The times required for the CNC-g-AA-2-120-8 and D-g-AA-2-120-8 aerogels to reach equilibrium are shown in Figure 4c. The swelling ratio of the CNC-g-AA-aerogel was almost nine times as great as that of the D-g-AA-2-120-8 aerogel at absorption equilibrium. We attribute this difference to the incorporation of CNCs into the gel matrix, which promoted the formation of a porous and honeycomb morphology. The CNCs supported the polymer network and provided sites for the initiation of polymerization, which opened up the aerogel structure and thus contributed to the absorption capacity and swelling rate of the hydrogels [42]. In addition, the presence of CNCs in the hydrogel matrix increased the amounts of hydrophilic groups, which enabled faster and easier diffusion of aqueous solution into the hydrogel matrix [43,44].

### 3.4. Formation Mechanism of CNC-g-AA Aerogels

**Figure 5:**
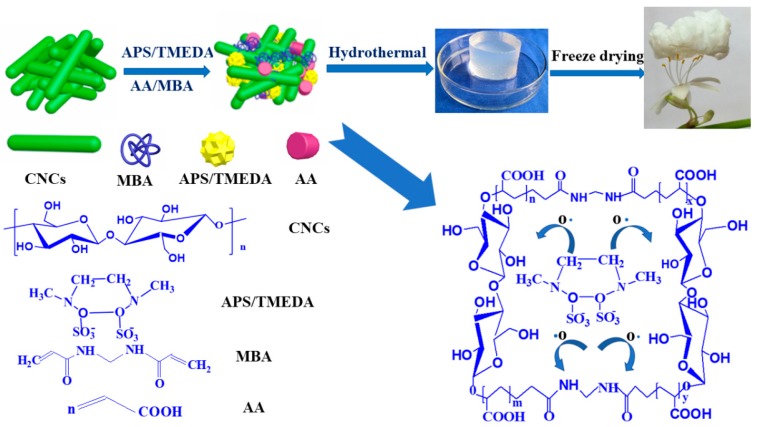
Mechanistic diagram of CNC-g-AA aerogel formation.

A proposed mechanistic pathway for the formation of CNC-g-AA aerogels is shown in Figure 5. Sulfate anion radicals generated from APS abstracted protons from –OH groups of the CNC backbone to form alkoxy radicals, which resulted in active centers on the CNCs backbone that radically initiated polymerization. The CNCs are rich in –OH groups, which induce strong molecular interactions in the nanocomposite system [45]. After AA is added to the nanocomposites the AA becomes conjugated with the CNCs and contributes more effective binding sites. The presence of the cross-linking reagent (MBA) results in the formation of a copolymer network, which comprises a chemical cross-linked structure [46]. During the hydrothermal treatment of the CNCs with the composites, the presence of –COO^−^, which is formed by deprotonation of –COOH groups, localizes the negative charge of the polymer network and enhances electrostatic repulsion, which favors expansion of the chain network. However, owing to the chemical composition of the CNC molecules, the attached –OH functional groups might induce hydrogen bond formation, which is an additional factor that improves adhesive properties [47].

### 3.5. Adsorption Studies

#### 3.5.1. Studies on Dye Adsorption

**Figure 6:**
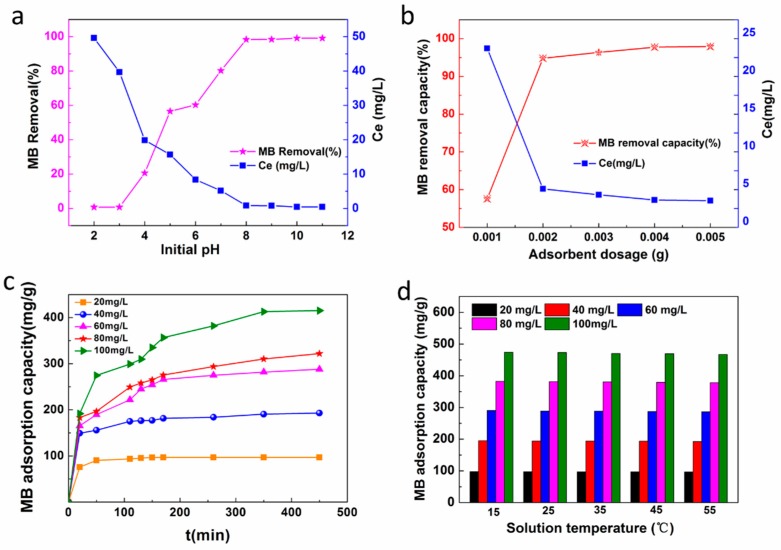
(**a**) Effects of solution pH on MB removal, (**b**) effects of adsorbent dosages on MB removal, (**c**) effects of contact time and initial MB solution concentration on MB adsorption, (**d**) effects of solution temperature on MB removal at different initial MB concentrations.

The effects of the solution pH of the initially prepared MB solutions on MB dye removal (%) and the residual concentration (C_e_) of the MB after adsorption by our CNC-g-AA-2-120-8 aerogels, are shown in Figure 6a. Here, we used a 0.0050-g portion of the CNC-g-AA-2-120-8 aerogel in 50 mL of a 60 mg/L MB solution. When the solution pH was decreased from 8.0 to 3.0, the ratio of MB removal decreased from 98.4% to 20.6% and the C_e_ decreased from 0.8 to 39.7 mg/L. We attribute the low adsorption ability at low pH to the CNC-g-AA-2-120-8 aerogels being immersed in an acidic environment having a more positive net charge at its surfaces, which electrostatically repelled MB.

To select a suitable amount of the adsorbent, different portions of (0.0010, 0.0020, 0.0030, 0.0040, and 0.0050 g) of CNC-g-AA-2-120-8 aerogels were added to 50 mL of 60 mg/L MB solutions, and the MB removal results are shown in Figure 6b. The adsorption capacity for the MB dyes on CNC-g-AA-2-120-8 aerogels decreased from 719.8–244.7 mg/g as the dosage increased from 0.0010 to 0.0050 g. However, the MB removal rate increased from 57.6% to 97.9% as the adsorbent dosage was increased from 0.0010 to 0.0050 g. The adsorption reached a swelling equilibrium when the adsorbent dosage was 0.0040 g. We attribute this result to the greater surface area of the adsorbent and the availability of more active adsorptive sites as the amount of the CNC-g-AA-2-120-8 aerogel was increased.

The initial MB concentration and the contact time with the CNC-g-AA-2-120-8 aerogels affected the adsorption capacity and removal efficiency. The experiments were performed at 25 °C, at pH = 8, with different initial concentrations (20–100 mg/L) and different contact times (20–450 min). The results are shown in Figure 6c. The adsorption capacity clearly increased from 96.98 to 415.13 mg/g as the initial MB concentration was increased from 20 to 100 mg/L. This result was attributed to the initial MB concentration providing the necessary driving force to surmount the resistance between the aqueous and solid phases and the increase of the initial MB concentration improved interactions between dye molecules and adsorbents, which provided heterogeneous adsorption sites [48,49].

The effects of solution temperature on MB removal by CNC-g-AA-2-120-8 aerogels are shown in Figure 6d. The MB adsorption capacity of the CNC-g-AA-2-120-8 aerogels decreased slightly with increasing temperature, from 15 to 55 °C at the same initial MB concentration. The MB adsorption capacity slightly decreased from 97.74 to 96.57 mg/g as the solution temperature was increased from 15 to 55 °C at a MB concentration of 20 mg/g. The MB adsorption capacity decreased from 474.22 to 467.31 mg/g at a MB concentration of 100 mg/g. The effects of temperature on MB adsorption might be attributed to the exothermic nature of the adsorption reaction. [50]

#### 3.5.2. Adsorption Kinetics

**Figure 7:**
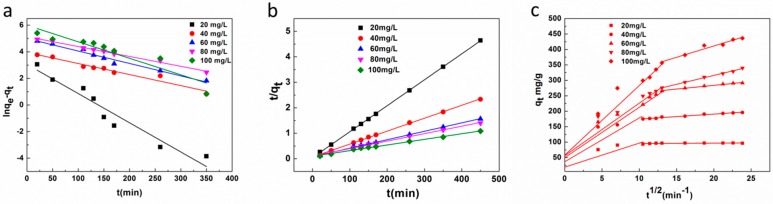
(**a**) Lagergren-first-order kinetic model, (**b**) pseudo-second-order kinetic model and (**c**) intra-particle diffusion model for adsorption of MB onto CNC-g-AA-2-120-8 aerogels at 25 °C.

The effects of the MB initial concentration and adsorption time on the CNC-g-AA-2-120-8 aerogels are shown in Figure 6c. The value of q_e_ increased rapidly during the initial stage of adsorption and then increased further at a relatively slow adsorption rate, before finally reaching equilibrium after approximately 260 min when the initial concentration was 100 mg/L. At an initial concentration of 40 mg/L, the aerogel reached swelling equilibrium (q_e_) after 100 min, beyond which there was almost no increase in adsorption. A higher initial concentration of the solution required a longer equilibrium swelling time. We used a pseudo-first-order and pseudo-second-order equations to analyze the mechanism of the adsorption process. These can be expressed in linear form as:

(3) $${\ln(q}_{e}\;-{\;q}_{t}{)\;=\;\mathrm{lnq}}_{e}-k_{1}t,$$

(4) $$\frac{t}{\mathrm{qt}}=\frac{1}{K_{2}q_{e}^{2}}+\frac{t}{q_{e}}$$

where q_e_ (mg/g) is the adsorption capacity of the CNC-g-AA-2-120-8 aerogels at equilibrium, K_1_ (min^−1^) is the rate constant of the pseudo-first-order model, and K_2_ (g /mg min^−1^) is the rate constant of the pseudo-second-order model. Equations (3) and (4) were used to fit the experimental data, as shown in Figure 7a,b respectively. The kinetic parameters obtained are summarized in Table 1. For all CNC-g-AA aerogels, the calculated correlation coefficients (R^2^) were closer to unity for the pseudo second-order kinetic model than for the pseudo first-order kinetic model. Therefore, these results indicated that the dye adsorption of the CNC-g-AA-2-120-8 aerogels was described well by a pseudo-second-order kinetic model.

We used the intra-particle diffusion model Equation (5) to further determine the mechanism of the nanocomposite gels for dye considering the diffusion of dye molecules:

(5) $$q_{t}={k_{i}}^{\frac{1}{2}}+C$$

where K_i_ is the intra-particle diffusion rate constant (mg/g min^1/2^) and C is a constant related to the extent of the boundary layer effect. As shown in Figure 7c, the kinetic data also agreed well with the Elovich model, further indicating the heterogeneous chemisorption character of the CNC-g-AA-2-120-8 aerogels. Moreover, intra-particle diffusion of dye molecules played an important role at the initial stage of adsorption, as suggested by the intra-particle diffusion model fitting well with our experimental kinetic data.

#### 3.5.3. Adsorption Isotherm and Thermodynamics

**Figure 8:**
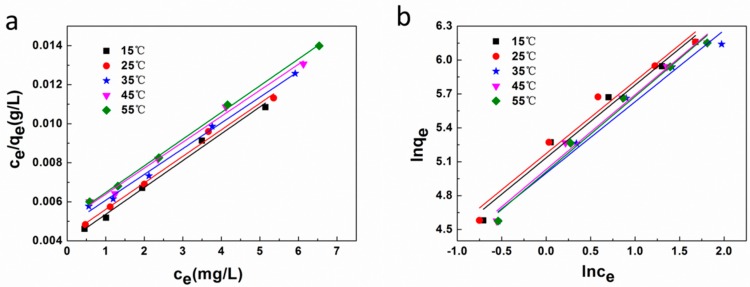
(**a**) Langmuir and (**b**) Freundlich isotherms for the adsorption of MB by CNC-g-AA-2-120-8 aerogels at different temperatures.

We determined the MB adsorption onto the CNC-g-AA-2-120-8 aerogels as a function of the equilibrium concentration of dye solution (C_e_, mg/L). Langmuir and Freundlich isotherm equations were used to determine the isotherm parameters and linear fitting based on the two isotherm models are shown in Figure 8a,b. The obtained isotherm parameters are summarized in Table 2. The Langmuir isotherm results suggested a surplus of atoms or molecules on the adsorbent surface, the effects of MB residual valences were comparable to the molecular diameter; hence, the Langmuir isotherms describes monolayer adsorption. The Langmuir isotherm model is valid for monolayer adsorption onto a surface with a finite number of identical and equivalent sites and can be expressed in the following linear form:

(6) $$\frac{C_{e}}{q_{e}}=\frac{1}{q_{m}k_{L}}+\frac{C_{e}}{q_{m}}$$

where q_m_ is the maximum dye adsorption onto adsorbent (mg/g) and K_L_ is the Langmuir adsorption equilibrium constant (L/g). The Freundlich isotherm model is an empirical equation that is regularly applied to multilayer adsorption, with a non-uniform distribution of adsorption heat and affinities over the heterogeneous surface of absorbent. It can be expressed in the following linear form:

(7) $$L_{n}q_{e}=L_{n}k_{F}+\frac{1}{n}L_{n}C_{e}$$

Three thermodynamic parameters must be considered during studies of adsorption processes, namely, enthalpy (∆H), and entropy (∆S). The values of ∆H and ∆S can be obtained by the van’t Hoff equation as follows:

(8) $${\mathrm{lnK}}_{d}=\frac{\Delta s}{R}-\frac{\Delta H}{\mathrm{RT}}$$

where R (8.314 J/mol K) is the universal gas constant, T (K) is the absolute temperature, and K_d_ is the equilibrium constant. The value of K_d_ can be calculated as:

(9) $$k_{d}=\frac{q_{e}}{C_{e}}$$

The value of K_d_ can be determined by measurements at different temperatures, where q_e_ and C_e_ are the equilibrium concentration of the dye on the adsorbent (mg/L) and in solution (mg/L), respectively. The values of ∆H and ∆S can be obtained from the slope and intercept of ln K_d_ versus 1/T, respectively, and the free energy change ∆G can be obtained from the following relation:

(10) $$\Delta G=-{\mathrm{RTl}}_{n}K_{C}$$

The ∆G, ∆H, and ∆S values of adsorption are shown in Table 3. The negative values of ∆H confirmed that the adsorption process was exothermic in nature. The spontaneous properties and feasibility of the adsorption via physical forces as well as the high tendency of the adsorbent for the adsorbate. Thus, lower temperatures promoted adsorption of MB, which is in accordance with the results of the adsorption isotherms.

#### 3.5.4. Adsorption and Regeneration Analysis

On the basis of the above experimental observations and our determination of the kinetic and equilibrium isotherm parameters, we propose a mechanism to describe the MB adsorption process and mechanism. Nanoscale networks with large open pores in the CNC-g-AA aerogels allowed the entry and rapid diffusion of ions and molecules, giving these materials good performance as adsorbents. The CNC-g-AA aerogel samples adsorbed MB dye through swelling and the penetration of dye into the CNC-g-AA aerogel network. The high adsorption capacity indicates strong electrostatic attractions between the dye molecules and sorbent binding sites. The surface of the CNCs and other introduced active groups also promoted electrostatic adsorption at chelating effects to remove dye molecules [51,52].

**Figure 9:**
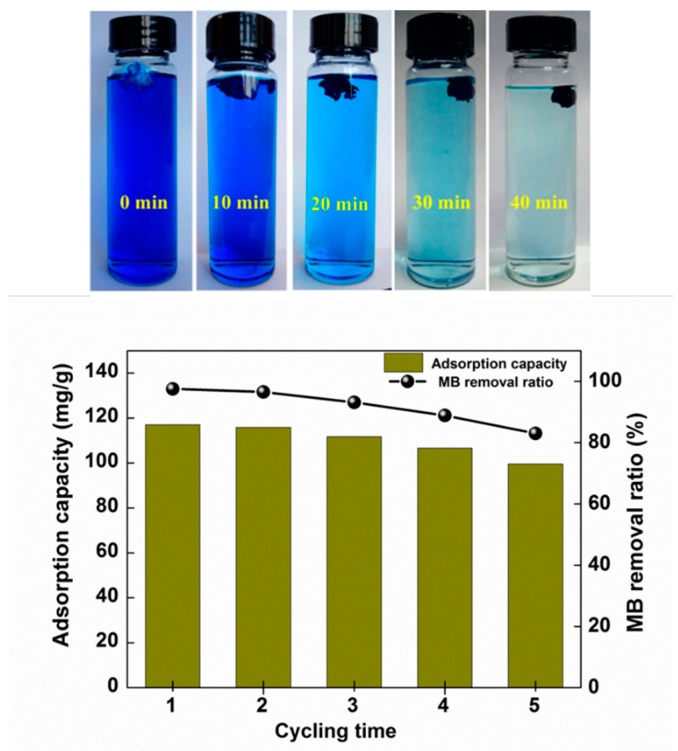
Adsorption and regeneration performance of CNC-g-AA-2-120-8 aerogels for MB.

The ability to regenerate an adsorbent is an important factor for evaluating its potential practical applications. The experimental results of adsorption-desorption regeneration are shown in Figure 9. The MB removal rate and the adsorption capacity clearly decreased obviously after three adsorption-desorption experiments. After five cycles the removal ratio of MB by CNC-g-AA-2-120-8 aerogels remained greater than 83.1% and the MB adsorption capacity remained greater than 99.7 mg/g This finding illustrates that the CNC-g-AA-2-120-8 aerogels can be reused as adsorbents for selective removal of dye pollution.

## 4. Conclusions

CNC-g-AA hydro/aerogels were successfully fabricated via a hydrothermal treatment followed by freeze drying. The swelling ratio of our CNC-g-AA aerogels was as high as 495, which was much higher than other polysaccharide-g-AA aerogels systems. Adsorption isotherms indicated that the MB adsorptive behavior onto CNC-g-AA aerogels followed the Langmuir model. The kinetics of the MB adsorption followed a pseudo second-order model, suggesting that the adsorption process was chemical adsorptions dominant. The kinetic data also agreed well with the Elovich model, which further indicated the heterogeneous chemisorption character. The maximum adsorption capacity of the CNC-g-AA aerogels on MB was greater than 400 mg/g and showed excellent regeneration ability. Our study demonstrated that the application of these CNC-g-AA aerogels can be extended to adsorption of dye contamination and agricultural residues from wastewater.

## Figures and Tables

**Table 1:** Adsorption kinetic parameters for MB adsorption of CNC-g-AA-2-120-8 aerogels.

C_0_ (mg/L)	q_e_,_exp_ (mg/g)	Lagergren-First-Order Kinetic Model			Pseudo-Second-Order Kinetic Mode		
		$q_{e1}$	K_1_ × 10^−3^ (1/min)	R^2^	$q_{e2}$	K_2_ × 10^−4^ (g/mg min)	R^2^
20	97.6388	96.9829	21.95	0.9240	98.1354	19.00	0.9999
40	194.8410	194.1255	8.31	0.9489	196.8505	5.50	0.9992
60	291.0470	290.5800	9.00	0.9591	302.1148	1.50	0.9972
80	383.0140	327.9173	7.43	0.9910	341.2970	1.00	0.9957
100	473.2217	416.6342	12.42	0.8629	452.4887	0.35	0.9926

**Table 2:** Adsorption isotherm parameters for MB adsorption of CNC-g-AA aerogels.

Langmuir				Frendlich		
T (°C)	K_L_ (L/mg)	q_max_ (mg/g)	R^2^	K_F_	n	R^2^
15	0.3467	724.6377	0.9903	169.8320	1.5524	0.9731
25	0.3200	735.2941	0.9914	176.3358	1.5596	0.9635
35	0.2773	757.5758	0.9925	148.2559	1.5818	0.9731
45	0.2628	751.8797	0.9912	153.2657	1.5139	0.9771
55	0.2681	729.9270	0.9980	150.1148	1.4996	0.9823

**Table 3:** Thermodynamic parameters for the adsorption of MB onto CNC-g-AA-2-120-8 aerogels.

Concentration (mg/L)	Temperature (℃)	K_d_	∆G (KJ/mol)	∆H (KJ/mol)	∆S (J/mol K)
20	15	216.7150	−12.8853		
	25	206.7594	−13.2160		
	35	166.6885	−13.1073	−12.4781	26.62
	45	166.6885	−13.5727		
	55	166.6885	−13.9580		
40	15	232.8024	−13.0569		
	25	197.1423	−13.0979		
	35	162.6617	−13.0447	−12.3480	22.73
	45	155.8044	−13.3540		
	55	146.9974	−13.6150		
60	15	149.0488	−11.8986		
	25	144.8095	−12.3332		
	35	136.4267	−12.5940	−11.6424	25.72
	45	122.1927	−12.7113		
	55	121.0599	−13.0854		
80	15	109.4590	−11.2490		
	25	104.1624	−11.5165		
	35	97.8559	−11.7427	−10.8564	25.72
	45	92.1381	−11.9645		
	55	91.1484	−12.3111		
100	15	92.2062	−10.8381		
	25	88.3592	−11.1086		
	35	79.5277	−11.2114	−10.5610	19.87
	45	76.6037	−11.4761		
	55	71.4717	−11.6477		
